# Supplementary material for: Genome-Wide Identification and Expression Analysis of TGA Family Genes Associated with Abiotic Stress in Sunflowers (Helianthus annuus L.)
Source: Int J Mol Sci. 2024 Apr 7;25(7):4097. doi: 10.3390/ijms25074097 (PMC11012525; doi:10.3390/ijms25074097)
Supplement: Supplementary file 1 [file ijms-25-04097-s001.zip › ijms-2932536-supplementary.pdf]

Supplementary Figures and Tables

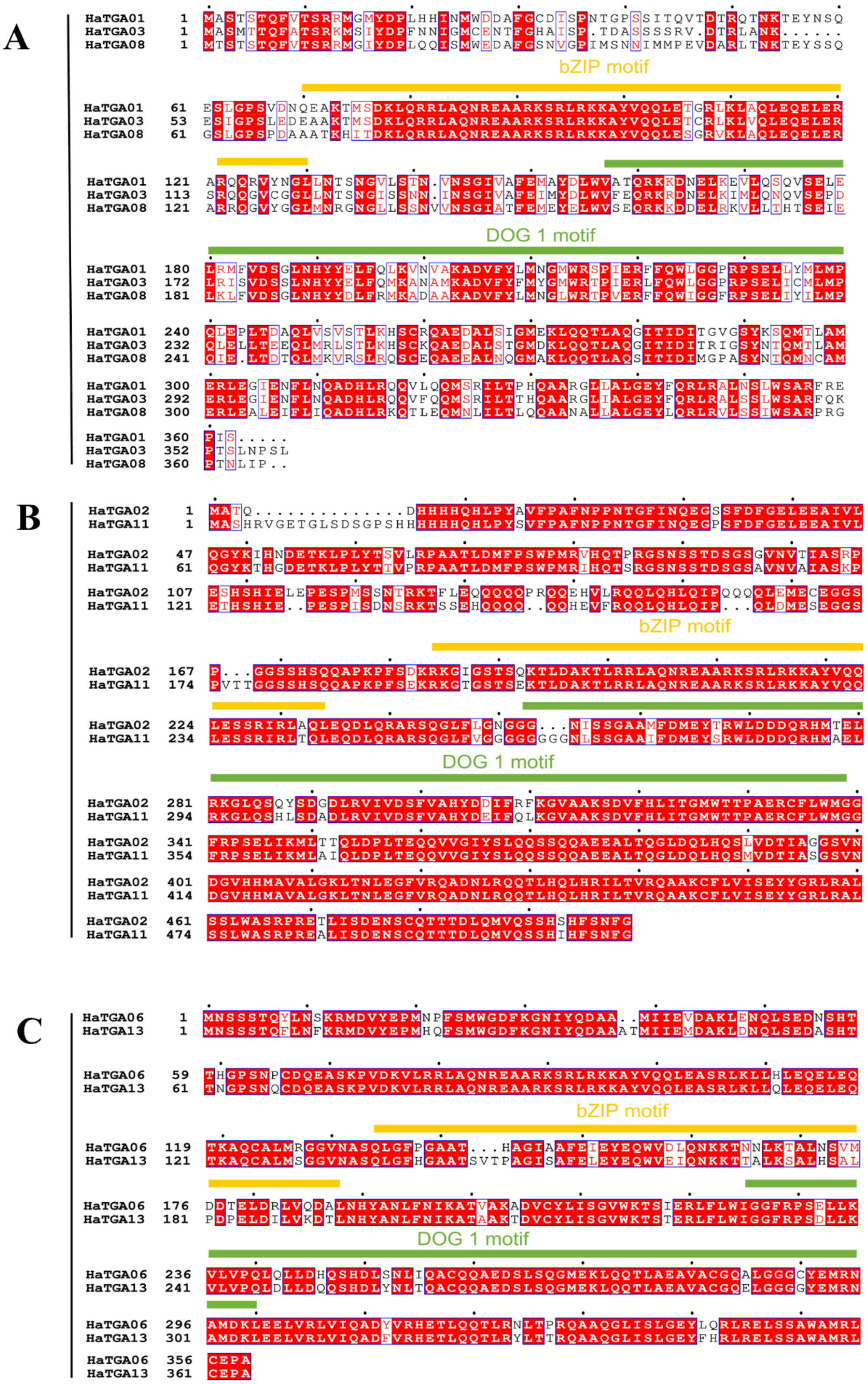

Figure S1. Protein sequence alignment analysis of closely related HaTGA. (**A**) Multiple sequence alignment of HaTGA01, HaTGA03, and HaTGA08, highlighting similarities and differences in their amino acid sequences. (**B**) Pairwise protein sequence alignment of HaTGA02 and HaTGA11 protein sequence. (**C**) Protein sequence conservation of HaTGA06 and HaTGA13. The protein sequence annotated by yellow and green lines indicated the presence of the bZIP and DOG1 domains, respectively.

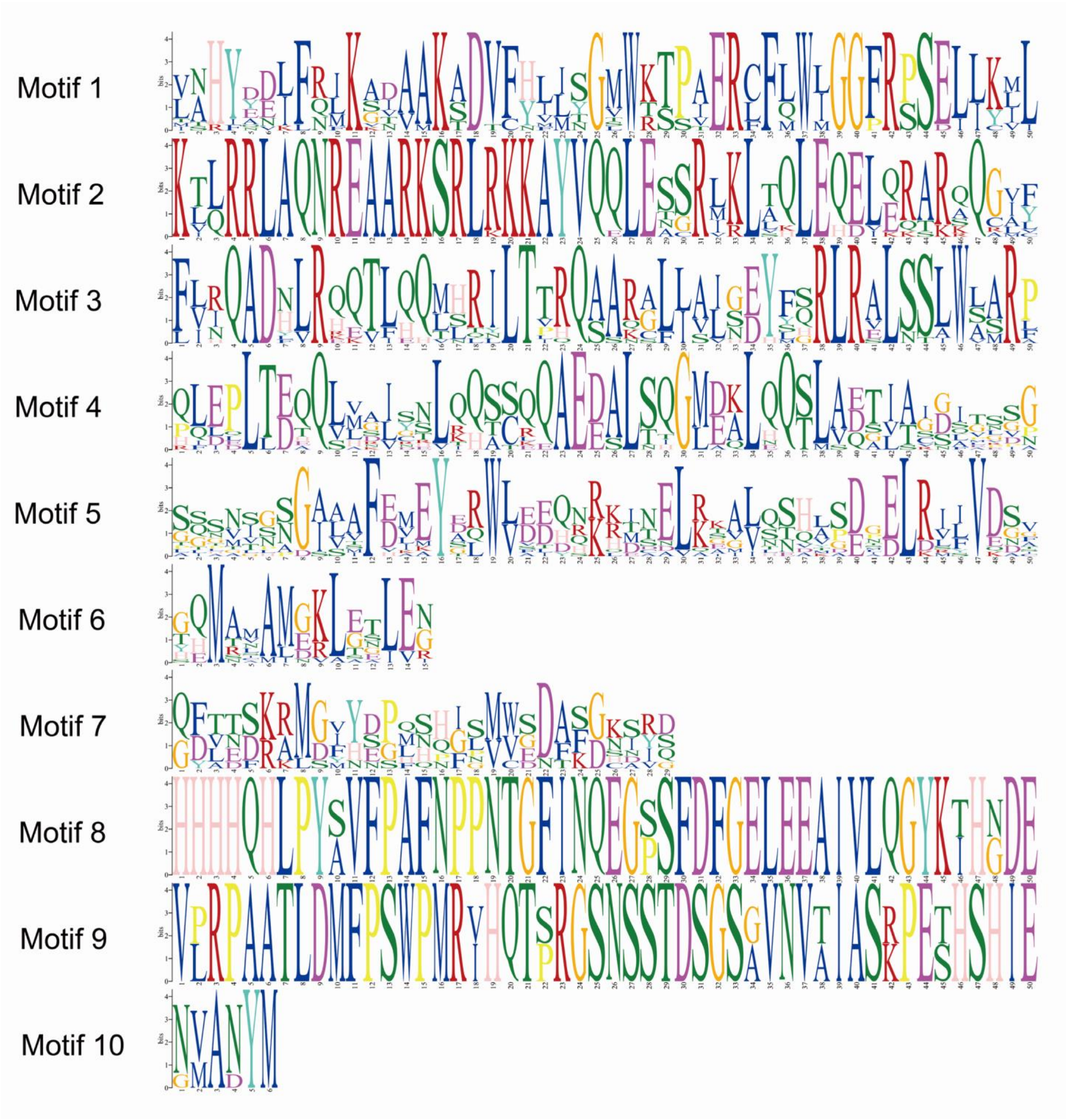

Figure S2. Visualization of conserved motifs within sunflower TGA proteins. Each logo consists of stacks of letters, with each letter representing an amino acid. The total height of the stacks at a specific position reflects the degree of conservation across the analyzed sequences. The height of an individual letter within the letter stacks indicates the relative frequency of the corresponding amino acid at that position.

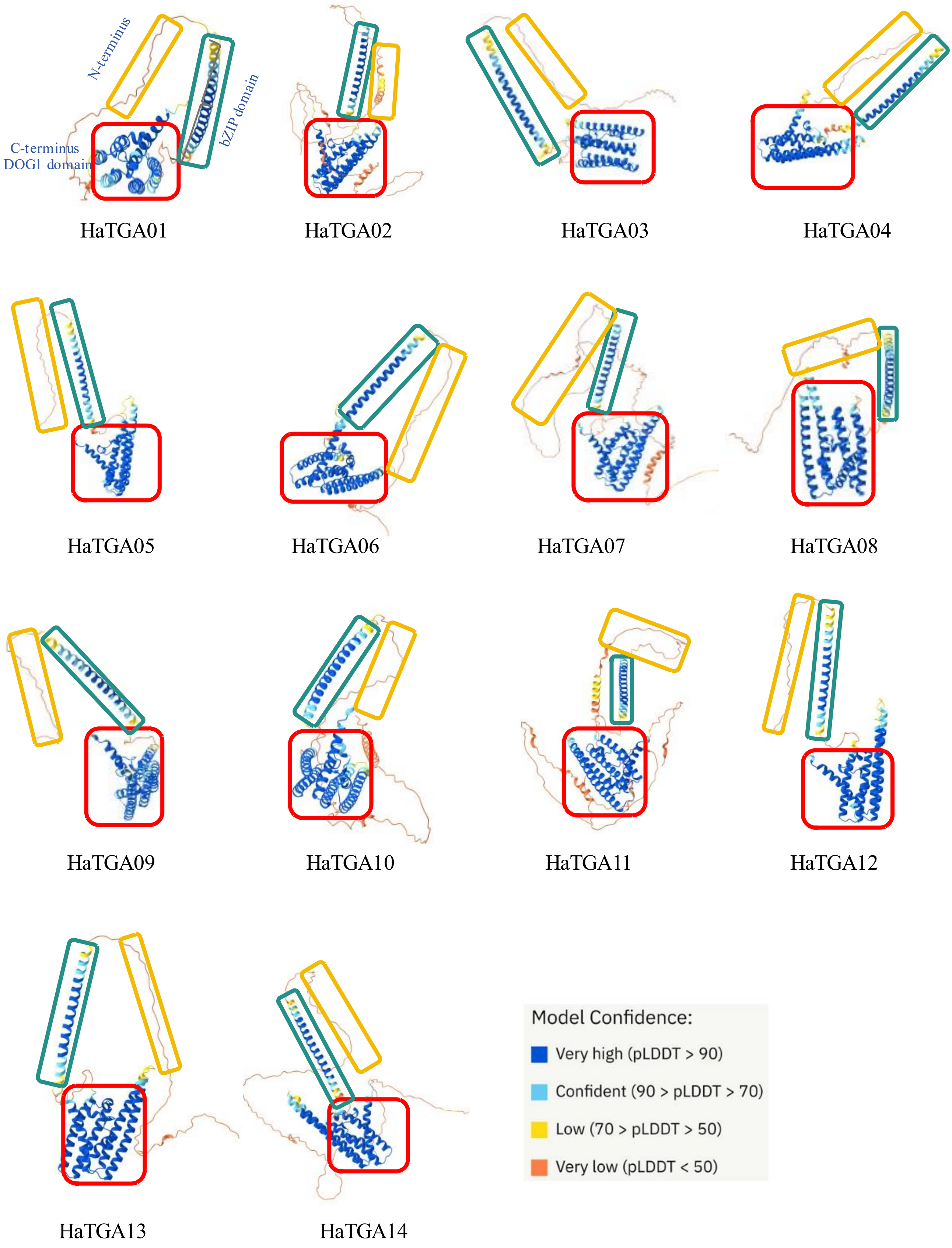

Figure S3. In Silico prediction of 3D structures for TGA proteins in sunflower. The predicted tertiary structures of TGA proteins from sunflowers were generated through AlphaFold computational modeling. As a schematic representation of the HaTGA01 domain, showing the flexible N-terminus (yellow dashed box), the bZIP domain (green dashed box), and the C-terminus, encompassing a putative Delay of Germination 1 (DOG1) domain (red dashed box). (pLDDT, AlphaFold per-residue confidence score).

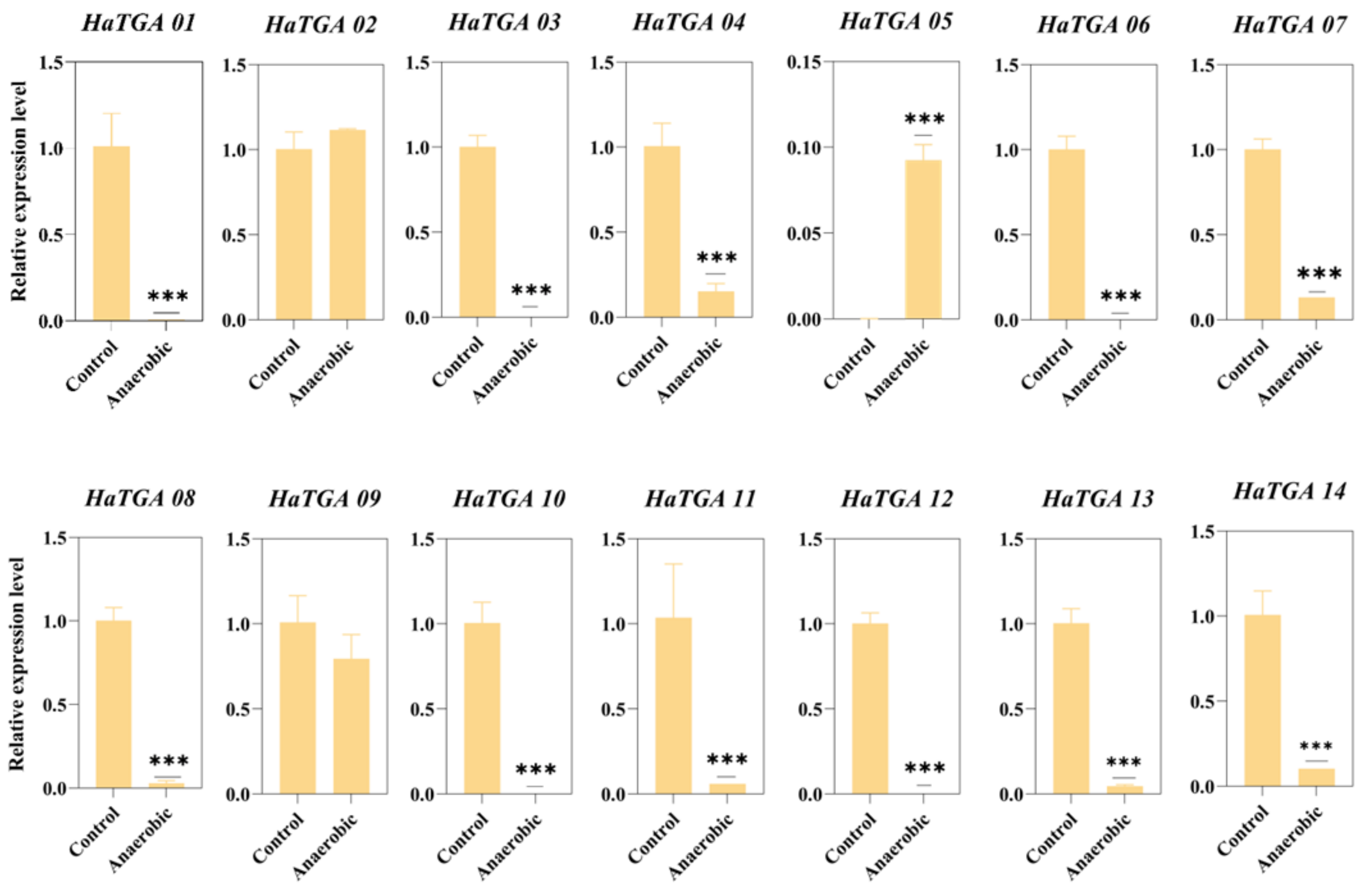

Figure S4. Expression profiles of *HaTGA* genes under anaerobic induction. The relative expression levels were calculated using the $2^{-\Delta\Delta Ct}$ method and compared with that of *HaTublin*, with three replicates. The values represent the means and standard deviations obtained from three biological replicates. The asterisks indicate statistical significance (*** $P < 0.001$) compared to the corresponding control.

Table S1. Molecular characteristics of TGA in 13 plant species.

Gene ID	Rename	Number of Amion Acids	MW(Da)	PI	Instability Index	Aliphatic Index	Grand Average of Hydropathicity
AT5G65210	AtTGA01	368	42063.57	7.28	37.3	76.63	-0.524
AT5G06950	AtTGA02	330	36684.29	8.63	52.04	79.36	-0.576
AT1G22070	AtTGA03	384	43755.04	5.85	59.79	76.25	-0.635
AT5G10030	AtTGA04	364	41751.28	6.68	42.57	75.6	-0.557
AT5G06960	AtTGA05	330	36890.39	8.58	57.32	79.03	-0.611
AT3G12250	AtTGA06	355	39716.66	8.92	54.04	79.8	-0.579
AT1G77920	AtTGA07	368	41913.06	5.97	74.16	84.35	-0.605
AT1G68640	AtTGA08	452	50457.51	5.85	51.61	77.7	-0.486
AT1G08320	AtTGA09	481	53514.28	7.82	64.63	76.94	-0.529
AT5G06839	AtTGA10	460	51166.56	6.7	62.94	78.96	-0.564
Phvul.001G123300	PvTGA01	460	50821.98	8.53	57.04	75.83	-0.55
Phvul.001G249300	PvTGA02	362	40912.63	8.41	52.91	79.06	-0.457
Phvul.003G028800	PvTGA03	462	51489.33	6.6	45.09	84.03	-0.463
Phvul.007G003600	PvTGA04	444	49112.79	6.18	56.88	76.1	-0.534
Phvul.007G025500	PvTGA05	462	51461.47	6.03	47.79	77.29	-0.493
Phvul.008G169000	PvTGA06	369	41753.53	6.38	57.21	83.33	-0.417
Phvul.009G026900	PvTGA07	351	39724.52	6.28	50.86	94.47	-0.312
Phvul.011G203400	PvTGA08	467	51713.7	6.13	55.14	76.55	-0.586
ArahyR4ID1P	AhTGA01	503	56631.81	6.69	64.38	68.73	-0.691
ArahyLTZY89	AhTGA02	469	52191.43	6.4	51.96	73.28	-0.564
ArahyS1DMKE	AhTGA03	469	52191.43	6.4	51.96	73.28	-0.564
ArahyRIV6ZB	AhTGA04	368	41626.26	8.34	55.67	76.44	-0.485
Arahy0GP62G	AhTGA05	388	43615.67	5.84	46.31	97.81	-0.248
Arahy661MCQ	AhTGA06	459	51238.71	6.21	44.32	79.98	-0.51
ArahyKZ9VWQ	AhTGA07	450	49513.54	7	56.22	78.18	-0.482
ArahyJ1CFJJ	AhTGA08	487	53623.35	7.38	50.59	72.36	-0.558
ArahyRKP385	AhTGA09	331	37018.61	8.63	58.5	80	-0.592
ArahyW15JJQ	AhTGA10	456	50921.93	6.31	44.7	79.23	-0.518
ArahyP1JBJN	AhTGA11	463	51147.27	6.53	56.06	81.19	-0.491
Arahy5F9GBV	AhTGA12	504	56713.86	6.56	64.22	68.79	-0.692
ArahyEH3L7F	AhTGA13	531	59169.39	6.49	55.39	73.92	-0.553
Arahy6K442L	AhTGA14	368	41586.2	8.34	56.08	76.71	-0.476
ArahyH6GQLR	AhTGA15	469	52078.53	5.97	43.48	78.68	-0.483
ArahyPHPT4F	AhTGA16	450	49483.51	7	56.22	78.4	-0.477
Arahy980X4K	AhTGA17	492	54382.57	8.27	45.32	81.14	-0.398
ArahySERN02	AhTGA18	331	37018.61	8.63	58.02	80	-0.592
ArahyK04A4E	AhTGA19	456	50968.95	6.34	46.34	78.38	-0.541
ArahyAAI19J	AhTGA20	362	41028.9	6.63	53.47	89.01	-0.384
Glyma01G084200	GmTGA01	486	54309.28	7.32	44.12	81.89	-0.47
Glyma02G097900	GmTGA02	467	51853.46	7.32	42.02	85.44	-0.469
Glyma02G176800	GmTGA03	484	53927.08	7.77	60.57	76.65	-0.538
Glyma03G127600	GmTGA04	460	50928	7	54.91	72.89	-0.586
Glyma03G128200	GmTGA05	290	32365.88	8.82	55.22	84.21	-0.336
Glyma03G142400	GmTGA06	491	55200.29	8.22	62.53	73.56	-0.659
Glyma04G254800	GmTGA07	362	40929	8.69	49.65	94.03	-0.338
Glyma05G182500	GmTGA08	370	42096.79	7.11	41.28	81.97	-0.468
Glyma06G107300	GmTGA09	355	39984.68	6.21	55.03	96.17	-0.286
Glyma08G140100	GmTGA10	379	43105.04	7.78	42.38	83.38	-0.466
Glyma10G092100	GmTGA11	517	57921.5	6.61	65.08	74.2	-0.579
Glyma10G276100	GmTGA12	456	50993.85	6.08	51.35	75.13	-0.518
Glyma10G296200	GmTGA13	332	37141.79	8.94	56.91	82.11	-0.563
Glyma11G183700	GmTGA14	493	54385.8	5.87	55.11	76.45	-0.502
Glyma11G236300	GmTGA15	364	41178.84	6.28	56.1	78.08	-0.464
Glyma12G088700	GmTGA16	501	55253.16	6.61	53.46	78.1	-0.463

Glyma12G184500	GmTGA17	488	54118.97	6.75	51.19	75.43	-0.501
Glyma13G085100	GmTGA18	370	41915.73	7.13	58.65	85.24	-0.461
Glyma13G193700	GmTGA19	469	51989.98	5.98	56.7	77.65	-0.587
Glyma13G316900	GmTGA20	490	54630.43	6.96	56.92	75.31	-0.531
Glyma14G167000	GmTGA21	370	41888.77	8.26	58.29	84.7	-0.452
Glyma15G232000	GmTGA22	497	55125.35	6.1	54.95	77.24	-0.639
Glyma18G020900	GmTGA23	362	41052.71	7.78	55.3	78.51	-0.487
Glyma19G130200	GmTGA24	459	50717.82	8.53	54.01	75.16	-0.574
Glyma19G145300	GmTGA25	491	55358.4	8.71	62.05	70.59	-0.715
Glyma20G113600	GmTGA26	455	50671.73	5.98	47.72	77.43	-0.447
Glyma20G246400	GmTGA27	444	49505.41	5.91	59.17	80.9	-0.537
LOC_Os01g17260	OsTGA01	329	36837.43	7.08	57.46	78.72	-0.638
LOC_Os01g59350	OsTGA02	472	51326.5	6.82	46.79	80.59	-0.431
LOC_Os01g64020	OsTGA03	539	59538.67	6.47	61.49	73.84	-0.536
LOC_Os02g10140	OsTGA04	303	31720.57	9.21	57.25	73.7	-0.39
LOC_Os03g20310	OsTGA05	333	37069.92	8.57	56.15	85.68	-0.486
LOC_Os04g54474	OsTGA06	397	45091.99	5.67	49.04	78.69	-0.565
LOC_Os05g37170	OsTGA07	540	59423.98	5.76	62.45	75.65	-0.358
LOC_Os06g15480	OsTGA08	385	42332.67	6.16	54.51	79.22	-0.366
LOC_Os06g41100	OsTGA09	451	49272.93	8.82	56.55	76.12	-0.399
LOC_Os07g48820	OsTGA10	334	37215	7.14	49.66	85.42	-0.495
LOC_Os08g07970	OsTGA11	417	45610.51	6.39	63.47	77	-0.416
LOC_Os09g10840	OsTGA12	475	52155.13	8.56	56.5	76.32	-0.512
LOC_Os09g31390	OsTGA13	523	57853.86	6.72	67.95	72.26	-0.578
LOC_Os11g05480	OsTGA14	430	47012.52	6.19	50.94	70	-0.568
LOC_Os12g05680	OsTGA15	489	52859.21	6.84	52.6	72.84	-0.438
Lsat_1_v5_gn_1_4841	LsTGA01	362	41054.64	7.78	44.67	85.25	-0.471
Lsat_1_v5_gn_2_31440	LsTGA02	330	36573.19	8.63	59.69	83.48	-0.511
Lsat_1_v5_gn_3_59901	LsTGA03	473	52435.66	6.28	45.37	74.5	-0.529
Lsat_1_v5_gn_4_139840	LsTGA04	348	39132.43	5.63	42.04	85.34	-0.442
Lsat_1_v5_gn_5_65440	LsTGA05	332	36972.5	8.65	60.63	81.81	-0.535
Lsat_1_v5_gn_5_157020	LsTGA06	430	47645.39	8.51	62.38	74.93	-0.546
Lsat_1_v5_gn_6_38701	LsTGA07	518	57739.51	6.79	54.49	73.46	-0.604
Lsat_1_v5_gn_6_102001	LsTGA08	326	37900.26	8.36	44.73	87.67	-0.534
Lsat_1_v5_gn_9_27821	LsTGA09	383	42502.29	8.97	66.43	81.33	-0.501
Lsat_1_v5_gn_9_43101	LsTGA10	366	41788.69	8.71	44.83	86.69	-0.459
SIN_1013641	SiTGA01	461	50346.52	6.4	46.75	80.63	-0.407
SIN_1017084	SiTGA02	371	41962.53	5.84	52.47	86.66	-0.427
SIN_1018321	SiTGA03	457	50049.16	6.81	54.96	83.46	-0.417
SIN_1018708	SiTGA04	433	47883.39	6.85	55.26	73.58	-0.599
SIN_1005924	SiTGA05	347	38402.35	9.4	60.18	85.62	-0.49
SIN_1023559	SiTGA06	538	59902.27	6.8	58.04	71.51	-0.577
SIN_1020070	SiTGA07	468	51572.68	8.52	52.14	73.25	-0.555
SIN_1009252	SiTGA08	383	43180.21	8.94	54.74	91.78	-0.333
SIN_1008334	SiTGA09	360	41017.72	7.16	54.46	87.31	-0.497
OIT30766	NaTGA01	372	41908.67	5.7	47.1	87.39	-0.36
OIT05628	NaTGA02	507	56322.16	6.48	59.56	73.81	-0.554
OIT05314	NaTGA03	454	49873.74	8.53	53.24	74.45	-0.578
OIT28278	NaTGA04	325	36261.85	9.01	64.79	84.8	-0.523
OIT03648	NaTGA05	495	54223.78	6.35	46.97	79.47	-0.435
OIT01585	NaTGA06	327	36500.22	8.96	62.73	84.25	-0.513
OIT30028	NaTGA07	501	55926.56	6.26	56.27	73.13	-0.577
OIS99738	NaTGA08	501	56249.09	6.41	67.92	75.25	-0.634
OIT23206	NaTGA09	363	41108.1	7.1	52.02	79.59	-0.382
OIS97611	NaTGA10	373	42301.05	6.19	42.23	82.71	-0.454
OIT04348	NaTGA11	514	57849.94	6.51	64.94	69.73	-0.678
OIS95689	NaTGA12	363	41240.01	7.02	54.16	75.84	-0.485
Sb010G110100	SbTGA01	462	50093.64	6.34	53.46	81.6	-0.21
Sb003G329500	SbTGA02	468	51130.35	6.39	55.59	77.5	-0.498
Sb003G081000	SbTGA03	330	36580.06	7.88	57.45	79.64	-0.583

Sb009G180700	SbTGA04	332	36982.68	6.34	58.95	84.22	-0.519
Zm00001d037317	ZmTGA01	432	46925.11	6.95	56.6	77.62	-0.268
Zm00001d043153	ZmTGA02	422	45869.22	5.91	49.9	71.78	-0.5
Zm00001d010658	ZmTGA03	345	38042.98	7.16	51.58	83.57	-0.508
Zm00001d012553	ZmTGA04	470	50719	6.34	46.71	77.79	-0.403
Zm00001d008225	ZmTGA05	420	46995.43	9.25	57.19	87.17	-0.416
Ca09600	CaTGA01	363	41395.08	5.48	55.68	87.38	-0.368
Ca11168	CaTGA02	356	40261.81	6.17	51.66	89.35	-0.377
Ca17971	CaTGA03	470	52026.14	6.29	52.93	79.55	-0.55
Ca25784	CaTGA04	475	56352.258	6.19	42.48	82.38	-0.453
Ca10901	CaTGA05	332	37207.91	8.63	61.43	80.66	-0.56
Ca05471	CaTGA06	373	41878.89	6.01	48.93	81.9	-0.458
Ca07170	CaTGA07	458	50956.13	7.85	57.94	74.24	-0.618
Ca12670	CaTGA08	384	43490.52	6.68	56.53	80.86	-0.447
VIT_01s0011g03230.t01	VvTGA01	349	39125.45	5.97	44.05	85.9	-0.375
VIT_06s0080g00360.t01	VvTGA02	361	41021.15	6.61	51.79	76.29	-0.592
VIT_07s0031g01320.t01	VvTGA03	469	51594.52	6.05	55.26	76.23	-0.535
VIT_08s0007g05170.t01	VvTGA04	451	49909.81	7.85	59.05	74.92	-0.576
VIT_08s0007g06160.t01	VvTGA05	423	46222.72	5.28	37.43	75.67	-0.356
VIT_13s0084g00660.t01	VvTGA06	491	54643.93	6.64	61.47	75.4	-0.549
VIT_18s0001g04470.t01	VvTGA07	500	54948.8	6.45	52.59	75.42	-0.459

Table S2. Genomic collinearity of *TGA* genes between sunflower and other species.

Chromosomal information of other species	The corresponding *TGA* gene	collinearity	Sunflower chromosome information	*HaTGA*
Gm-11	*GmTGA14*	==	Ha-2	*HaTGA02*
Gm-12	*GmTGA17*	==	Ha-2	*HaTGA02*
Gm-12	*GmTGA16*	==	Ha-2	*HaTGA02*
Gm-13	*GmTGA20*	==	Ha-2	*HaTGA02*
Gm-6	*GmTGA09*	==	Ha-2	*HaTGA01*
Gm-6	*GmTGA09*	==	Ha-4	*HaTGA03*
Gm-13	*GmTGA18*	==	Ha-5	*HaTGA04*
Gm-6	*GmTGA09*	==	Ha-5	*HaTGA04*
Gm-10	*GmTGA11*	==	Ha-10	*HaTGA07*
Gm-19	*GmTGA25*	==	Ha-10	*HaTGA07*
Gm-2	*GmTGA03*	==	Ha-10	*HaTGA07*
Gm-3	*GmTGA06*	==	Ha-10	*HaTGA07*
Gm-8	*GmTGA10*	==	Ha-11	*HaTGA08*
Gm-11	*GmTGA14*	==	Ha-13	*HaTGA11*
Gm-12	*GmTGA16*	==	Ha-13	*HaTGA11*
Gm-12	*GmTGA17*	==	Ha-13	*HaTGA11*
Gm-13	*GmTGA20*	==	Ha-13	*HaTGA11*
Gm-19	*GmTGA24*	==	Ha-13	*HaTGA10*
Gm-1	*GmTGA01*	==	Ha-17	*HaTGA14*
Gm-10	*GmTGA12*	==	Ha-17	*HaTGA14*
Gm-2	*GmTGA02*	==	Ha-17	*HaTGA14*
Gm-20	*GmTGA26*	==	Ha-17	*HaTGA14*
Ah-2	*AhTGA02*	==	Ha-2	*HaTGA02*
Ah-7	*AhTGA07*	==	Ha-2	*HaTGA02*
Ah-7	*AhTGA08*	==	Ha-2	*HaTGA01*
Ah-12	*AhTGA12*	==	Ha-2	*HaTGA02*
Ah-16	*AhTGA16*	==	Ha-2	*HaTGA01*
Ah-17	*AhTGA17*	==	Ha-2	*HaTGA02*
Ah-7	*AhTGA08*	==	Ha-5	*HaTGA04*
Ah-16	*AhTGA16*	==	Ha-5	*HaTGA04*
Ah-1	*AhTGA01*	==	Ha-10	*HaTGA07*
Ah-5	*AhTGA06*	==	Ha-10	*HaTGA07*
Ah-11	*AhTGA10*	==	Ha-10	*HaTGA07*
Ah-15	*AhTGA15*	==	Ha-10	*HaTGA07*
Ah-2	*AhTGA02*	==	Ha-13	*HaTGA11*
Ah-7	*AhTGA06*	==	Ha-13	*HaTGA11*
Ah-12	*AhTGA10*	==	Ha-13	*HaTGA11*
Ah-17	*AhTGA15*	==	Ha-13	*HaTGA11*
Ah-4	*AhTGA06*	==	Ha-17	*HaTGA14*
Ah-8	*AhTGA10*	==	Ha-17	*HaTGA14*
Ah-18	*AhTGA15*	==	Ha-17	*HaTGA14*
Ls-1	*LsTGA01*	==	Ha-2	*HaTGA01*
Ls-6	*LsTGA07*	==	Ha-2	*HaTGA02*
Ls-9	*LsTGA10*	==	Ha-2	*HaTGA01*
Ls-1	*LsTGA01*	==	Ha-4	*HaTGA03*
Ls-9	*LsTGA10*	==	Ha-4	*HaTGA03*
Ls-1	*LsTGA01*	==	Ha-5	*HaTGA04*
Ls-6	*LsTGA08*	==	Ha-5	*HaTGA04*
Ls-9	*LsTGA10*	==	Ha-5	*HaTGA04*
Ls-2	*LsTGA02*	==	Ha-6	*HaTGA05*
Ls-4	*LsTGA04*	==	Ha-9	*HaTGA06*
Ls-9	*LsTGA09*	==	Ha-10	*HaTGA07*
Ls-1	*LsTGA01*	==	Ha-11	*HaTGA08*

Ls-9	*LsTGA10*	==	Ha-11	*HaTGA08*
Ls-5	*LsTGA05*	==	Ha-12	*HaTGA09*
Ls-5	*LsTGA06*	==	Ha-13	*HaTGA10*
Ls-6	*LsTGA07*	==	Ha-13	*HaTGA11*
Ls-4	*LsTGA04*	==	Ha-15	*HaTGA13*
Ls-3	*LsTGA03*	==	Ha-17	*HaTGA14*
Si-9	*SiTGA09*	==	Ha-2	*HaTGA02*
Si-4	*SiTGA05*	==	Ha-10	*HaTGA07*
Si-5	*SiTGA06*	==	Ha-10	*HaTGA07*
Si-4	*SiTGA04*	==	Ha-13	*HaTGA10*
Si-9	*SiTGA09*	==	Ha-13	*HaTGA11*
Si-1	*SiTGA01*	==	Ha-17	*HaTGA14*
Si-2	*SiTGA03*	==	Ha-17	*HaTGA14*
Ca-1	*CaTGA02*	==	Ha-2	*HaTGA02*
Ca-6	*CaTGA01*	==	Ha-2	*HaTGA02*
Ca-3	*CaTGA07*	==	Ha-10	*HaTGA07*
Ca-4	*CaTGA06*	==	Ha-10	*HaTGA07*
Ca-1	*CaTGA02*	==	Ha-13	*HaTGA11*
Ca-6	*CaTGA01*	==	Ha-13	*HaTGA11*
Ca-4	*CaTGA05*	==	Ha-17	*HaTGA14*
Vv-18	*VvTGA07*	==	Ha-2	*HaTGA01*
Vv-6	*VvTGA02*	==	Ha-2	*HaTGA02*
Vv-8	*VvTGA04*	==	Ha-10	*HaTGA07*
Vv-18	*VvTGA07*	==	Ha-11	*HaTGA08*
Vv-6	*VvTGA02*	==	Ha-13	*HaTGA11*
Vv-1	*VvTGA01*	==	Ha-17	*HaTGA14*
At-1	*AtTGA03*	==	Ha-5	*HaTGA04*
At-5	*AtTGA10*	==	Ha-10	*HaTGA07*
At-1	*AtTGA09*	==	Ha-13	*HaTGA11*
At-1	*AtTGA08*	==	Ha-17	*HaTGA14*
Sb-3	*SbTGA02*	==	Ha-2	*HaTGA02*
Sb-10	*SbTGA01*	==	Ha-10	*HaTGA07*
Sb-3	*SbTGA02*	==	Ha-13	*HaTGA11*
Sb-9	*SbTGA04*	==	Ha-13	*HaTGA11*
Zm-3	*ZmTGA02*	==	Ha-2	*HaTGA02*

Table S3. qRT-PCR primer sequence information.

Primer name	Primer sequence (5'-3')
HaTublin-qRCR-F	CTGATTGTCGTAAACGCTTG
HaTublin-qRCR-R	CTCAAGATCAGCAACAGTGC
HaTGA01-qPCR-F	CCAGTGGCTTGGAGGACC
HaTGA01-qPCR-R	TGCATCCTCGGCTTGTCG
HaTGA02-qPCR-F	TATGGCGGTTGCGCTAGG
HaTGA02-qPCR-R	TCGCTGCTTGTCGAACCG
HaTGA03-qPCR-F	GCTTGGAGGACCACGACC
HaTGA03-qPCR-R	CCTCGGCTTGTTTGCACG
HaTGA04-qPCR-F	CGGATGCTGCGAAAACCG
HaTGA04-qPCR-R	TGATGGTCGAAACCCGCC
HaTGA05-qPCR-F	TGTCGCAGGGCATGGAAG

HaTGA05-qPCR-R	GCTACGTTGCCCGACGAA
HaTGA06-qPCR-F	GGCGTACGTCCAGCAGTT
HaTGA06-qPCR-R	AGCATTGACCCCACCACG
HaTGA07-qPCR-F	ATGGCGATGGGCCTTATGAT
HaTGA07-qPCR-R	ACTCGAACCCTCTCCAGTAGT
HaTGA08-qPCR-F	GCATGTGGGAGGATGCGT
HaTGA08-qPCR-R	TTTTGTTGCCGCAGCGTC
HaTGA09-qPCR-F	CTCAGCAGGCAGAGGACG
HaTGA09-qPCR-R	GGACCAATGGAGCCTGCT
HaTGA10-qPCR-F	ACTTGGAACCGCTGACCG
HaTGA10-qPCR-R	CCACGGGTGCATCTCTGG
HaTGA11-qPCR-F	GCCGAGCTGCGTAAAGGA
HaTGA11-qPCR-R	ACGTCGGATTTGGCTGCA
HaTGA12-qPCR-F	CTCGGCAGCAAGGCATCT
HaTGA12-qPCR-R	TGTGTTCGTCCAGCCACC
HaTGA13-qPCR-F	CAAGCATGCCAGCAAGCC
HaTGA13-qPCR-R	ACCGCCACCCAACTCTTG
HaTGA14-qPCR-F	ACTGAGGCAGCAAACGCT
HaTGA14-qPCR-R	GGCGCGCTAACCACAATG
